# Influence of Scanning Speed on Microstructure and Properties of Laser Cladded Fe-Based Amorphous Coatings

**DOI:** 10.3390/ma12081279

**Published:** 2019-04-18

**Authors:** Xiangchun Hou, Dong Du, Baohua Chang, Ninshu Ma

**Affiliations:** 1 State Key Laboratory of Tribology, Department of Mechanical Engineering, Tsinghua University, Beijing 100084, China; houxc16@mails.tsinghua.edu.cn (X.H.); dudong@tsinghua.edu.cn (D.D.); 2 Joining and Welding Research Institute, Osaka University, 11-1 Mihogaoka, Ibaraki, Osaka 567-0047, Japan; ma.ninshu@jwri.osaka-u.ac.jp

**Keywords:** Fe-based amorphous coating, laser cladding, microstructure, property

## Abstract

Fe-based amorphous alloys with excellent mechanical properties are suitable for preparing wear resistant coatings by laser cladding. In this study, a novel Fe-based amorphous coating was prepared by laser cladding on 3Cr13 stainless steel substrates. The influence of scanning speeds on the microstructures and properties of the coatings was investigated. The microstructure compositions and phases were analyzed by scanning electron microscope, electron probe microanalyzer, and x-ray diffraction respectively. Results showed that the microstructures of the coatings changed significantly with the increase of scanning speeds. For a scanning speed of 6 mm/s, the cladding layer was a mixture of amorphous and crystalline regions. For a scanning speed of 8 mm/s, the cladding layer was mainly composed of block grain structures. For a scanning speed of 10 mm/s, the cladding layer was composed entirely of dendrites. Different dilution rates at the bonding zones were the main reasons for the microstructure change for different claddings. For all three scanning speeds, the coatings had higher hardness and wear resistance when compared with the substrate; as the scanning speed increased, the hardness and wear resistance of the coatings gradually decreased due to the change in microstructure.

## 1. Introduction

Amorphous alloys have unique properties such as high hardness, high strength, high wear resistance, and high corrosion resistance because of their short-range order and long-range disordered structure [1,2,3]. Their unique properties make amorphous alloys suitable for surface coatings. Different processes have been used to prepare surface coatings such as laser cladding, plasma spray, spark cladding, and high velocity oxygen fuel (HVOF) [4,5,6,7,8]. Among them, laser cladding can meet the requirements of preparing amorphous alloys thanks to its rapid cooling characteristics. In addition, the laser cladded coatings may have good metallurgical bonding with substrates [9,10]. Therefore, laser cladding has attracted more and more attention in the fabrication of amorphous surface coatings. Since Yoshioka et al. [11] successfully deposited a Ni-Cr-P-B amorphous coating by laser cladding on low carbon steel in 1975, different types of amorphous alloy coatings have been prepared by laser cladding such as Zr-based, Ni-based, Cu-based, Al-based, Co-based, etc. [12,13,14,15,16]. Compared with the above amorphous alloy systems, Fe-based amorphous coatings have comparable mechanical properties but a much lower cost [17], and therefore have great potential in engineering applications. So far, efforts have been made to prepare different kinds of Fe-based amorphous coatings using laser cladding. Sahasrabudhe et al. [10] deposited Fe-Cr-Mo-W-C-Mn amorphous coatings on a Zr substrate by laser cladding where the coating had an amorphous and crystalline composite structure and the crystal phase was distributed in the amorphous region. The coatings had much higher hardness and wear resistance than the substrate. Zhu et al. [18] laser cladded Fe-Co-B-Si-Nb coatings on low carbon steel at different scanning speeds of 6, 17, and 50 m/s and found that the main factors that affected the microstructure and the fraction of the amorphous phase were dilution ratio and scanning speed. Wu et al. [19] prepared Fe-Co-Ni-Zr-Si-B amorphous coatings on AISI 1045 steel by laser cladding where the thickness of the coating was 1.2 mm and had a high hardness of 1270 HV as well as good corrosion resistance.

In the present paper, an Fe-based amorphous alloy with the chemical composition of ${\mathrm{Fe}}_{46.8}{\mathrm{Mo}}_{22.7}{\mathrm{Cr}}_{13.6}{\mathrm{Co}}_{7.6}C_{4.8}B_{2.3}Y_{1.2}{\mathrm{Si}}_{1.0}$ (wt.%), which had high glass forming ability, was used to prepare coatings by laser cladding. To our knowledge, the powders have not previously been used in laser cladding. The main objective of this study was to investigate the effects of scanning speeds on microstructure and properties of the coatings.

## 2. Materials and Methods

### 2.1. Materials

3Cr13 stainless steel with dimensions of 80 mm × 50 mm × 6 mm was used as the substrate material. The chemical composition of 3Cr13 stainless steel is shown in Table 1. The chemical composition of the Fe-based amorphous alloy powders was ${\mathrm{Fe}}_{46.8}{\mathrm{Mo}}_{22.7}{\mathrm{Cr}}_{13.6}{\mathrm{Co}}_{7.6}C_{4.8}B_{2.3}Y_{1.2}{\mathrm{Si}}_{1.0}$ (wt.%). Pure elemental powders of Fe, Mo, Cr, Co, C, Y, Si, and B with 99.8 to 99.9 wt.% in purity were used to prepare powders by vacuum gas atomization. The particle size of the powders was 40–75 μm.

### 2.2. Laser Cladding

A fiber laser (YLS-2000 IPG) equipped with a lateral powder feeder system was used in laser cladding. The laser beam with a Gaussian energy distribution was 3 mm in diameter. Ar gas was employed to transport powders and protect the bath from oxidation. The gas flow rate was 10 L/min. The laser cladding was carried out at the scanning speeds of 6, 8 and 10 mm/s. The detailed laser parameters used in this study are listed in Table 2.

### 2.3. Microanalysis

The laser cladding samples were cut along cross sections with a wire electric discharge machining (EDM) machine, then were polished and etched by aqua regia for microscopy observations and hardness tests. A scanning electron microscope (SEM, QUANTA 200 FEI, Hillsboro, OR, USA) with energy dispersive spectroscopy (EDS) and an electron probe microanalyzer (EPMA SHIMADZU, Kyoto, Japan) was used to analyze the microstructure and element distributions. The phase composition of the laser-processed samples were characterized by an x-ray diffractometer (D8 Bruker, Billerica, MA, USA) with Cu–Ka (λ = 0.154060 nm) radiation. The X-ray diffraction (XRD) system was operated at 40 kV and 200 mA in a 2θ range of 20–80°. The step size was 0.02° and the scanning speed was 4° per minute. Before the XRD test, the laser cladding samples with dimensions of 8 mm × 8 mm × 6 mm were ground to a position of 0.3 mm in a thickness direction from the top surface. 

### 2.4. Microhardness and Tribological Tests

The microhardness of the samples was measured using a Vickers hardness tester (HM-800 Mitutoyo, Takatsu-ku, Kawasaki, Japan) under a load of 200 g and a dwell time of 15 s. The samples with dimensions of 15 mm × 15 mm × 6 mm were cut from the laser cladding samples, and then were ground to keep the surface roughness less than Ra3.2. A ball-on-disc tribometer (UMT-3 Bruker, Billerica, MA, USA) was used to measure the tribological properties of the substrate and the coatings. A silicon nitride ball with a diameter of 4 mm and hardness of 1550 ${\mathrm{HV}}_{0.1}$ was used as the upper specimen. The wear experiment was performed at a load of 3 N and a sliding speed of 100 r/min, in a circle with the diameter of 10 mm for 30 min. A precision analytical balance with an accuracy of 0.001 mg was used to measure the wear mass losses and then calculate the volume losses. The worn morphologies and compositions were measured by scanning electron microscopy (SEM, QUANTA 200 FEI) with energy dispersive spectroscopy (EDS FEI).

## 3. Results and Discussion

### 3.1. Phases

**Figure 1:**
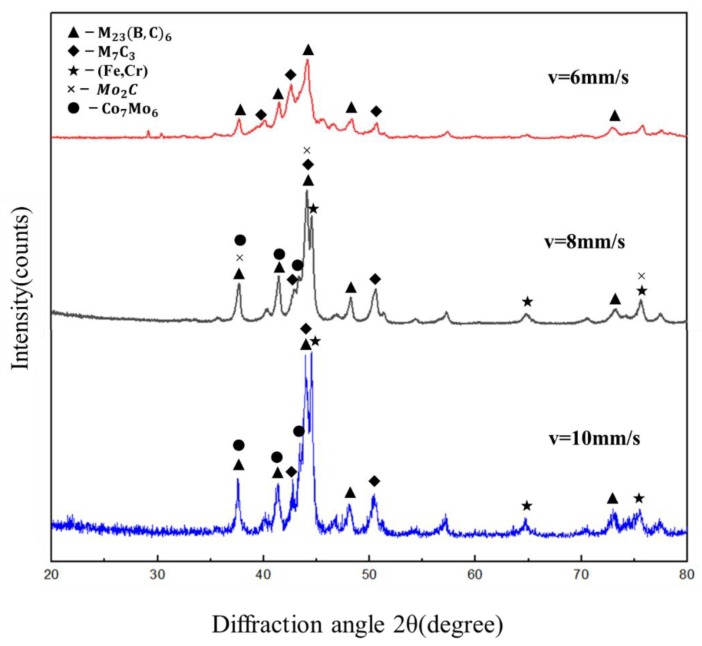
XRD patterns of the cladding layers at different scan speeds.

The x-ray diffraction patterns of the coatings produced at different laser scanning speeds are shown in Figure 1. The diffraction patterns varied significantly with the laser scanning speed. When the scanning speed was 6 mm/s, a broad halo peak characterizing an amorphous phase could be observed at the diffraction angle around 44°, on which sharp diffraction peaks corresponding to the carbides $M_{23}{\left(B,C\right)}_{6}$ and $M_{7}C_{3}$ superposed. Here, M is Fe and Cr. When the scanning speed was increased to 8 mm/s and 10 mm/s, the broad halo peak was no longer apparent and the diffraction patterns were composed of sharp peaks of crystalline phases. As the scanning speed increased, the intensity of the crystallization peak increased significantly, indicating an increase in crystallinity and a suppression of amorphous formation. There was a definite change in the phase constituents when the scanning speed was increased from 6 mm/s to 8 mm/s. Besides the carbides $M_{23}{\left(B,C\right)}_{6}$, $M_{7}C_{3}$, and ${\mathrm{Mo}}_{2}C$, diffraction peaks from the solid solution γ-(Fe,Cr) and intermetallic compound ${\mathrm{Co}}_{7}{\mathrm{Mo}}_{6}$ were also identified. The phases at the scanning speed of 10 mm/s were mainly carbides $M_{23}{\left(B,C\right)}_{6}$, $M_{7}C_{3}$, solid solution γ-(Fe,Cr), and intermetallic compound ${\mathrm{Co}}_{7}{\mathrm{Mo}}_{6}$, which were the same as those for 8 mm/s.

### 3.2. Geometrical Morphologies and Microstructures

**Figure 2:**
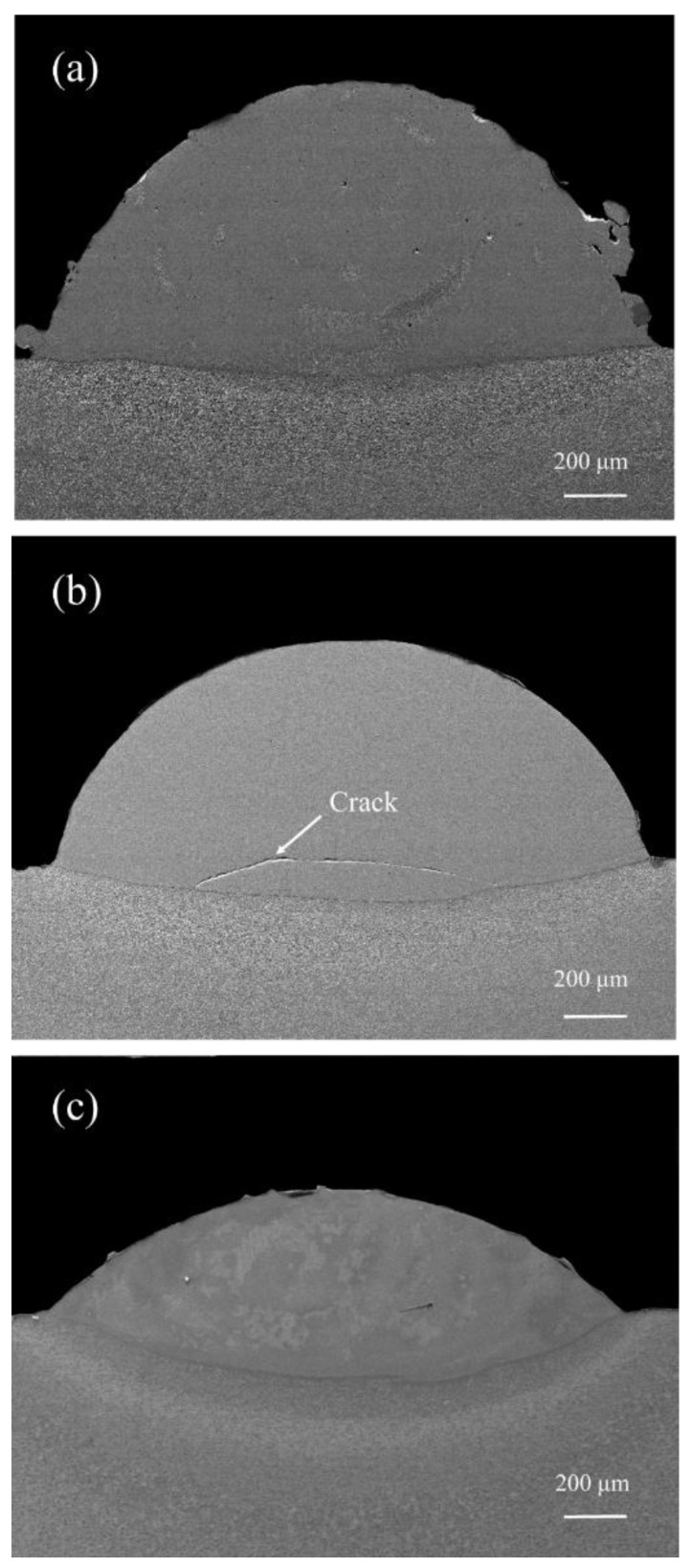
Cross sections of the laser cladding coatings for different scanning speeds: (**a**) 6 mm/s; (**b**) 8 mm/s; (**c**) 10 mm/s.

The cross sections of coatings obtained at different laser scanning speeds are shown in Figure 2. The cladding layers were dense and clear interface lines could be seen between the cladding layers and the substrates for all three different scanning speeds, which indicated good metallurgical bonding was achieved in laser cladding. Cracks may have existed in some cladding layers (Figure 1). The curvature of the interface decreased as the heat input increased, indicating the change in dilution rates. The geometric parameters on cross-sections of coatings at different scanning speeds are shown in Table 3. The dilution rates were calculated using Equation (1) [20].

(1) $$\mathrm{Dilution}\;\mathrm{rate}\;\mu =\frac{S_{2}}{S_{1}+S_{2}}\times 100\%,$$

where $S_{1}$ is the area of cladding layer and $S_{2}$ represents the area of molten substrate on the cross-section of a specimen. The average geometric parameters and the dilution rates are presented in Table 3.

The width and height of the cladding layers decreases with an increase in scanning speeds under comparable laser cladding conditions because the amount of powder fed into the molten pool per unit length is reduced as the scanning speed increases. On the other hand, increasing scanning speed leads to an increase in the depth of the melted substrate, and in turn, the dilution rate. This can be attributed to the weaker “heat shielding effect” [21] of the powders and more heat absorbed by the substrates at higher scanning speeds.

**Figure 3:**
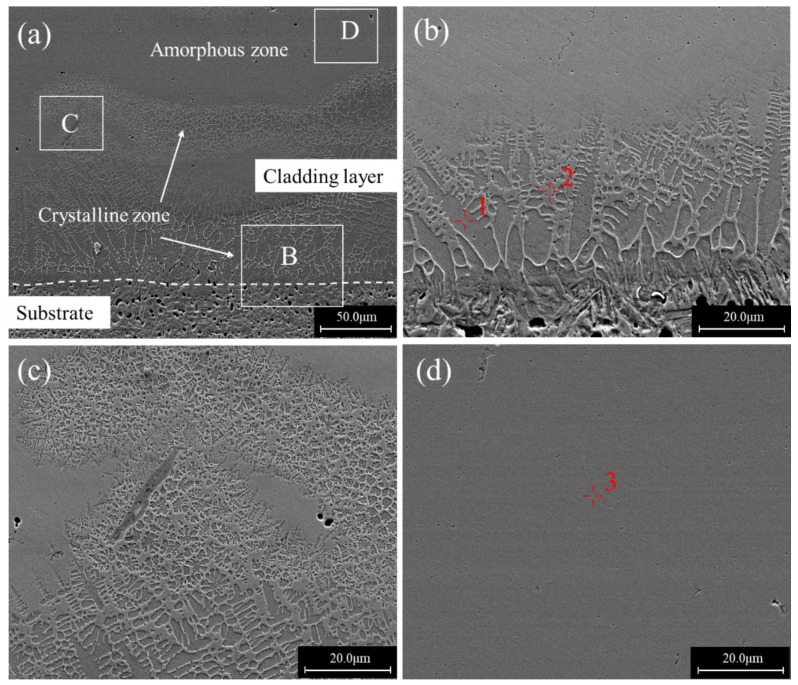
Cross sectional morphologies of a single-bead cladded specimen produced at the scanning speed of 6 mm/s: (**a**) Overall view; (**b**) an enlarged view of region B in (**a**); (**c**) an enlarged view of region C in (**a**); (**d**) an enlarged view of region D in (**a**).

**Figure 4:**
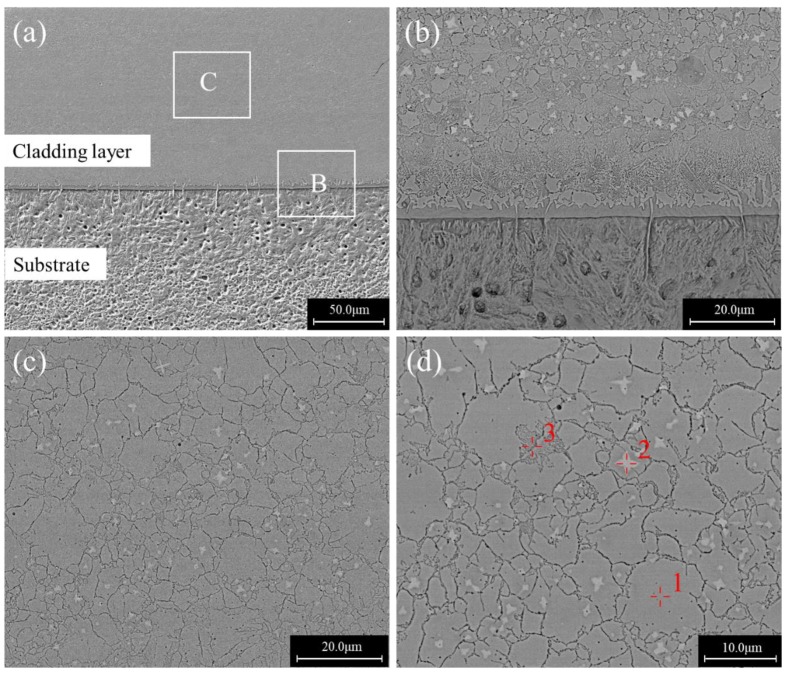
Cross sectional morphologies of a single-bead cladded specimen produced at the scanning speed of 8 mm/s: (**a**) Overall view; (**b**) an enlarged view of region B in (**a**); (**c**) an enlarged view of region C in (**a**); (**d**) an enlarged view of (**c**).

**Figure 5:**
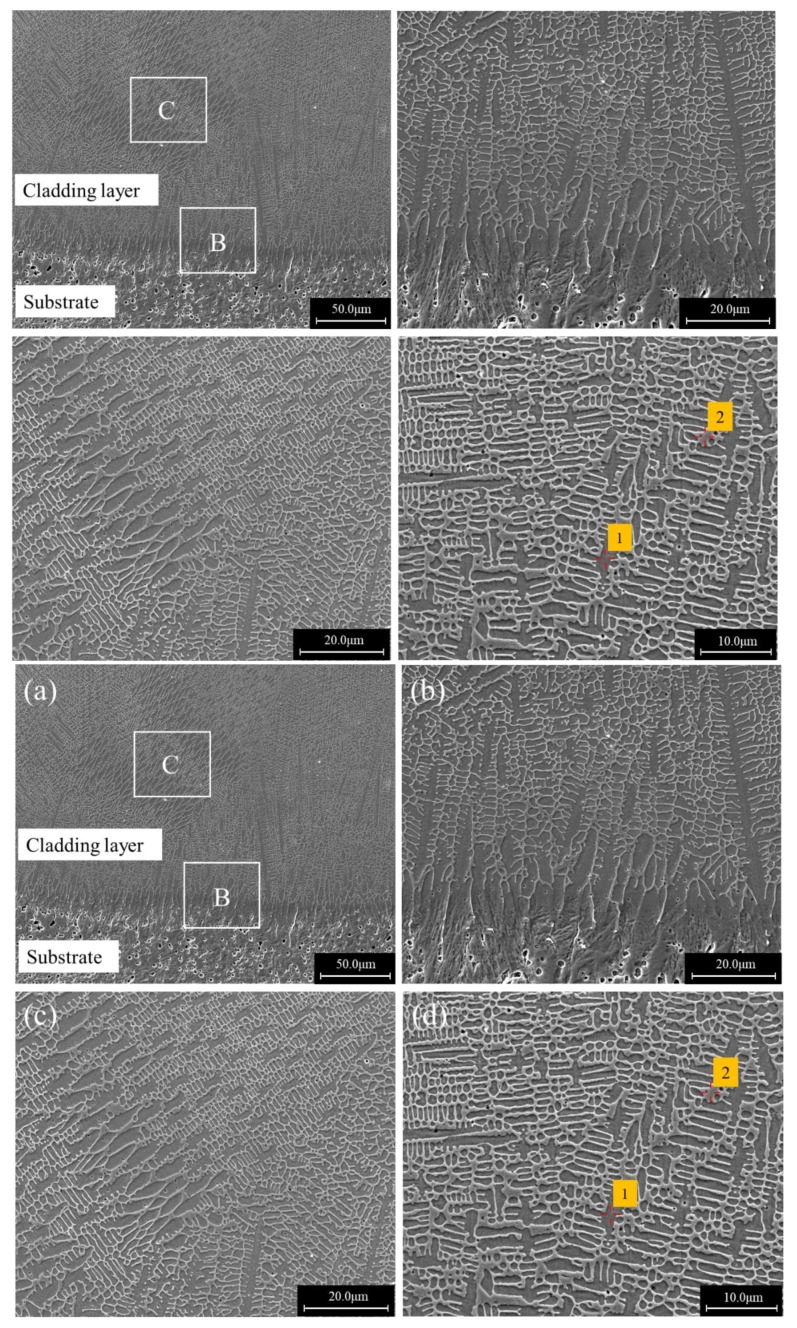
Cross sectional morphologies of a single-bead cladded specimen produced at the scanning speed of 10 mm/s: (**a**) Overall view; (**b**) an enlarged view of region B in (**a**); (**c**) an enlarged view of region C in (**a**); (**d**) an enlarged view of (**c**).

The typical microstructures of coatings made at different scanning speeds are presented in Figure 3, Figure 4 and Figure 5. Figure 3 shows the SEM photographs of the laser cladding for a scanning speed of 6 mm/s. Figure 3a is an overall view of the cross section, where the cladding layer shows a layered structure. The substrate under the interface exhibited a martensite structure, and a columnar crystal zone with a thickness of about 40–60 μm formed above the interface as shown in Figure 3b. Strip-shaped precipitates could be observed between columnar grains. Figure 3c is a magnification of area C, and it can be seen that above the columnar crystal region was a fine equiaxed crystal region surrounded by a featureless phase structure. In the upper part of the cladding layer, all of the microstructures were the featureless structure shown in Figure 3d. 

EPMA analysis revealed that the featureless phase structure (Point 3 in Figure 3d) had an average composition of ${\mathrm{Fe}}_{47.3}{\mathrm{Mo}}_{22.5}{\mathrm{Cr}}_{13.9}{\mathrm{Co}}_{7.2}C_{5.1}B_{2.2}Y_{1.0}{\mathrm{Si}}_{0.8}$ (wt.%), which was very close to the nominal composition of the powder. Combined with the XRD results, it can be inferred that this featureless structure zone was mainly composed of the amorphous phase. The EPMA at point 1 showed that the primary arm of the columnar dendrites at the bottom of the cladding layer had a composition of ${\mathrm{Fe}}_{81.5}{\mathrm{Mo}}_{1.2}{\mathrm{Cr}}_{11.6}{\mathrm{Co}}_{1.3}C_{2.6}B_{0.3}Y_{1.3}{\mathrm{Si}}_{0.5}$, where a large amount of Fe element was detected. The composition of the strip-shaped precipitates between columnar grains at Point 2 was ${\mathrm{Fe}}_{63.9}{\mathrm{Mo}}_{11.9}{\mathrm{Cr}}_{18.5}{\mathrm{Co}}_{2.4}C_{5.7}B_{2.0}Y_{0.2}{\mathrm{Si}}_{0.3}$ (wt.%), which had a relatively high carbon content and could mainly be Fe, Cr carbides. There was a higher Fe content and lower Mo, Cr contents in the columnar crystal region near the substrate than those in the upper amorphous region, which demonstrates the elements’ migration caused by the dilution effect.

The microstructures of the specimen with a scanning speed of 8 mm/s is shown in Figure 4. Figure 4a is an overall view of the cross section. Figure 4b is an enlarged backscattered photograph around the bonding region of the cladding layer and the substrate. There was a martensite structure under the interface between the cladding layer and the substrate, above which was a layer of planar crystal. Above the plane crystal was a layer of dendrites with a thickness of about 20–30 μm. Above the dendritic structure, the coating was composed of a block grain structure as shown in Figure 4c. Figure 4d is a further enlargement of the block grain structure in Figure 4c.

EPMA results showed that the chemical compositions of point 1 inside the block grain in Figure 4d was ${\mathrm{Fe}}_{47.4}{\mathrm{Mo}}_{22.6}{\mathrm{Cr}}_{15.5}{\mathrm{Co}}_{7.6}C_{5.6}B_{2.4}Y_{0.1}{\mathrm{Si}}_{0.6}$ (wt.%), which was close to the nominal composition of Fe-based amorphous alloy powder. The composition of the block grain boundary at point 3 was ${\mathrm{Fe}}_{44.9}{\mathrm{Mo}}_{22.9}{\mathrm{Cr}}_{14.6}{\mathrm{Co}}_{8.4}C_{7.5}B_{0.5}Y_{0.5}{\mathrm{Si}}_{0.7}$ (wt.%), which had a higher C content than the inside. In Figure 4d, a star-like white phase could be observed with a composition of ${\mathrm{Fe}}_{33.0}{\mathrm{Mo}}_{38.5}{\mathrm{Cr}}_{11.3}{\mathrm{Co}}_{6.8}C_{8.2}B_{1.8}Y_{0.1}{\mathrm{Si}}_{0.3}$ (wt.%) (Point 2), having high Mo and C content. Combined with the XRD phase analysis results, it can be inferred that it could be mainly composed of ${\mathrm{Mo}}_{2}C$ and $M_{23}{\left(B,C\right)}_{6}$. 

**Figure 6:**
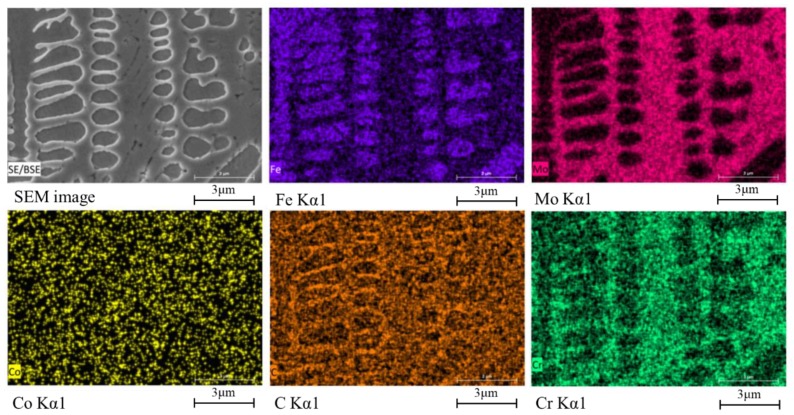
Element distributions of the cladding layer obtained at the scanning speed of 10 mm/s.

Figure 5 shows the SEM photographs of the specimen obtained at the scanning speed of 10 mm/s. It can be noted that dendritic structures existed throughout the cladding layer. In the vicinity of the interface between cladding and substrate, the columnar crystals grew upwards, and were perpendicular to the interface because the temperature gradient is large near the bonding zone, and columnar crystals grow along the temperature gradient direction [22]. It can also be seen that a large number of network-like precipitates existed between the dendrites. Figure 6 shows the elemental distributions obtained by EDS map scanning. It can be observed that the precipitates between the dendrites were rich in Mo, Cr, and C. In contrast, the dendrites were rich in Fe and C, with a trace of Cr, and a lack of Mo. There was no significant difference in the content of Co between the dendrites and the inner precipitates.

EPMA point scanning results showed that the chemical compositions of the dendrites at point 1 in Figure 6 was ${\mathrm{Fe}}_{76.7}{\mathrm{Mo}}_{6.9}{\mathrm{Cr}}_{14.7}{\mathrm{Co}}_{2.8}C_{3.5}B_{0.5}Y_{0.2}{\mathrm{Si}}_{0.8}$ (wt.%), which was dominated by Fe and Cr, with traces of Mo, C, and a little B. The chemical composition of the precipitates at point 2 in Figure 6 was ${\mathrm{Fe}}_{52.4}{\mathrm{Mo}}_{19.3}{\mathrm{Cr}}_{15.0}{\mathrm{Co}}_{3.5}C_{6.5}B_{1.9}Y_{0.8}{\mathrm{Si}}_{0.6}$ (wt.%). Combined with the XRD phase analysis results, it can be inferred that the dendrites were mainly γ-(Fe, Cr) solid solution and the network-like precipitates were composed of carbides $M_{23}{\left(B,C\right)}_{6}$ and $M_{7}C_{3}$. Zeisig et al. obtained a similar carbide network precipitation when laser cladding the Fe-Cr-Mo-V-C alloy [23].

In summary, as the scanning speeds increased, the microstructures of the laser cladding layers exhibited three distinct microstructure characteristics. For the scanning speed of 6 mm/s, the cladding layer was a mixture of amorphous and crystalline regions. When the scanning speed was increased to 8 mm/s, the cladding layer was mainly composed of a block grain structure. As the scanning speed was further increased to 10 mm/s, the cladding layer was composed entirely of dendrites. Combined with the XRD test results, it can be found that the glass forming ability decreases as the scanning speed increases. A large number of studies have shown that there are two main factors affecting the ability to form amorphous phases, i.e., the composition and the cooling rate [24,25,26]. The absence of amorphous structures can be attributed either to a cooling rate experienced by the coated layer, which is lower than the critical value required to form a glassy microstructure, or to a substantial solute redistribution or compositional changes within the molten pool or across the coating-substrate interface by dissolution or inter-diffusion. 

**Figure 7:**
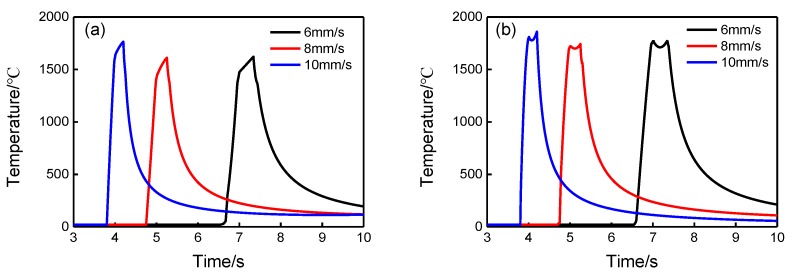
Thermal histories of points at: (**a**) deposit/substrate interface; (**b**) top surface of the deposits for the three scanning speeds obtained by finite element analysis.

The cooling rate ∂T/∂t during the laser cladding process can be approximately estimated using the Rosenthal solution proposed by Steen and Mazumder [27]:

(2) $$\frac{\partial T}{\partial t}=2\pi k\left[\frac{v}{P}\right]{\left(T-T_{0}\right)}^{2},$$

where *k* is thermal conductivity; *v* is scanning speed; *P* is power density; and $T_{0}$ is the initial temperature of the substrate. From the formula, it can be inferred that the cooling rate increases as the scanning speed increases. For a more quantitative study, the cooling rates were numerically computed in the present study using the finite element software JWRIAN developed by JWRI [28,29]. Figure 7 presents the thermal histories of points at the deposit/substrate interface and at the top surface of deposits for three scanning speeds. At the deposit/substrate interface, the cooling rates from 1000 °C to 500 °C for the three scanning speeds (6 m/s, 8 m/s, and 10 m/s) were 819.7, 1087.0, and 1388.9 °C/s, respectively. At the top surface of the deposits, the cooling rates from 1000 °C to 500 °C for the three scanning speeds were 847.5, 1111.1, and 1388.9 °C/s, respectively. Obviously, at both locations, the predicted cooling rates increased for the increased scanning speeds, which was the same as the analytical solution. Hence, increasing scanning speeds is theoretically favorable to the formation of an amorphous structure. Jin et al. [30] experimentally demonstrated that a higher scanning speed favored the formation of an amorphous phase in their work. However, the results in this study showed the opposite to the above deduction, where an amorphous microstructure tended to form at relatively lower scanning speed (6 mm/s) while dendrites formed at a higher speed (10 mm/s). Therefore, the main factor affecting the microstructures and amorphous forming ability of the deposits is more likely to be the change in the composition, but not scanning speed. As the laser scanning speed increased from 6 mm/s to 10 mm/s, the dilution ratios of the cladding layers increased significantly (by more than four times) as presented in Table 2. Consequently, more elements migrated from the substrates to the cladding layers, causing the compositions of the cladding layers to deviate from the nominal compositions of the Fe-based amorphous alloy powder, resulting in a decrease in the ability to form an amorphous phase. Li et al. [20] also showed that a coating with a lower dilution rate exhibited a larger volume fraction of the amorphous phase.

### 3.3. Microhardness

**Figure 8:**
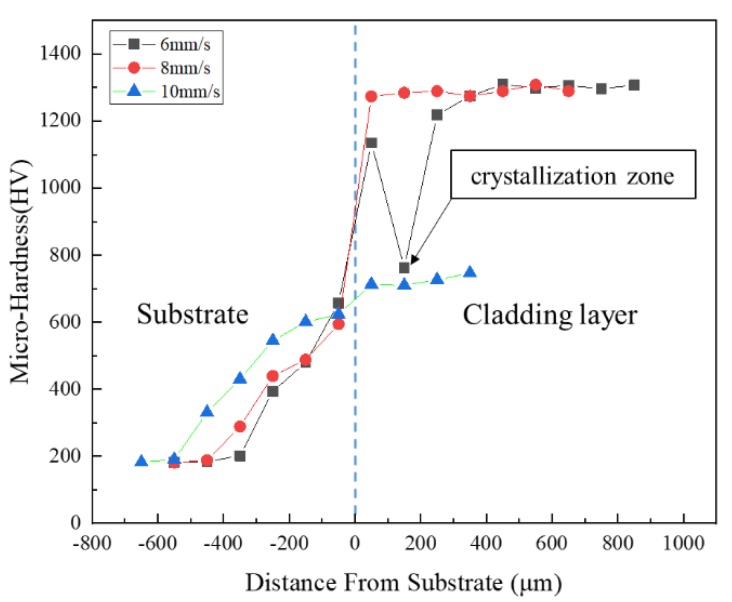
Variation of microhardness in the thickness direction of laser claddings produced with different scanning speeds.

The hardness distributions in the thickness direction for the cladding layers with different scanning speeds are shown in Figure 8. It can be found that the hardness decreased from the cladding layer to the heat affected zone and then to the substrate for all three scanning speeds. The cladding layer had an extremely high hardness of about 1300 ${\mathrm{HV}}_{0.2}$ for the scanning speeds 6 mm/s and 8 mm/s, and a lower high hardness of 700 ${\mathrm{HV}}_{0.2}$ for 10 mm/s. When the scanning speed was 6 mm/s, there was a sudden decrease in hardness to about 700 ${\mathrm{HV}}_{0.2}$ at 200 μm from the substrate, which was because the test point was located just in the crystallization zone in the cladding layer. Overall, the hardness of the cladding layers was significantly improved when compared to the substrate, and the hardness distribution in the cladding layer was quite stable. Both the amorphous phase and the block grain structure had extremely high hardness. The high hardness of the block grain structure formed at 8 mm/s may mainly be attributed to the formation of ${\mathrm{Mo}}_{2}C$ and other carbides [31].

### 3.4. Wear Resistance

**Figure 9:**
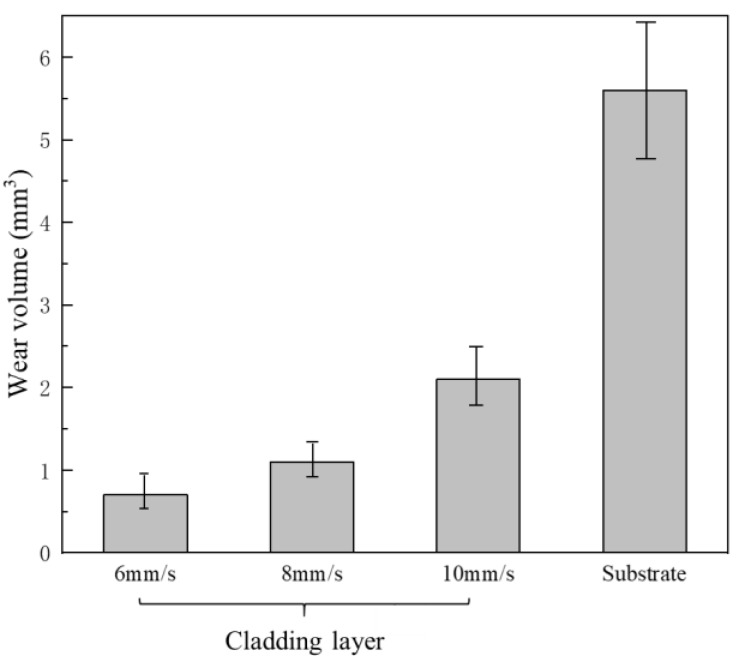
Volume losses in the wear tests for the substrate and the cladding layers produced with different scanning speeds.

Figure 9 shows the wear volume losses of the substrate and the coatings obtained at different scanning speeds. It is clear that the wear volume losses of the cladding layers increased gradually with an increase of the scanning speeds. The wear volume losses of coatings at the three scanning speeds were much smaller than that of the substrate, indicating that the cladding layers significantly improved the wear resistance of the substrate. With the scanning speed of 6 mm/s, the cladding layer had the smallest wear volume loss of 0.71 mm^3^, while that for the substrate was 5.60 mm^3^. The wear resistance of the substrate was increased by 7.9 times. 

**Figure 10:**
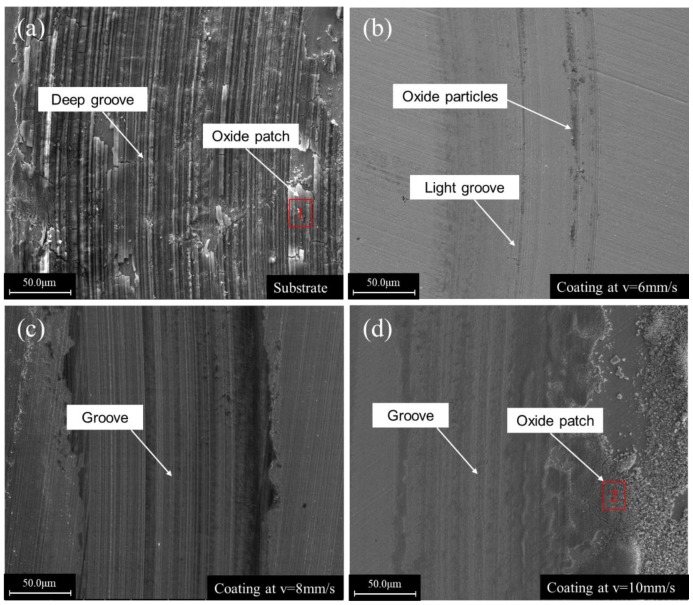
SEM micrographs of the worn surfaces of the substrate and the claddings obtained with different scanning speeds: (**a**) Substrate; (**b**) cladding at 6 mm/s; (**c**) cladding at 8 mm/s; (**d**) cladding at 10 mm/s.

**Figure 11:**
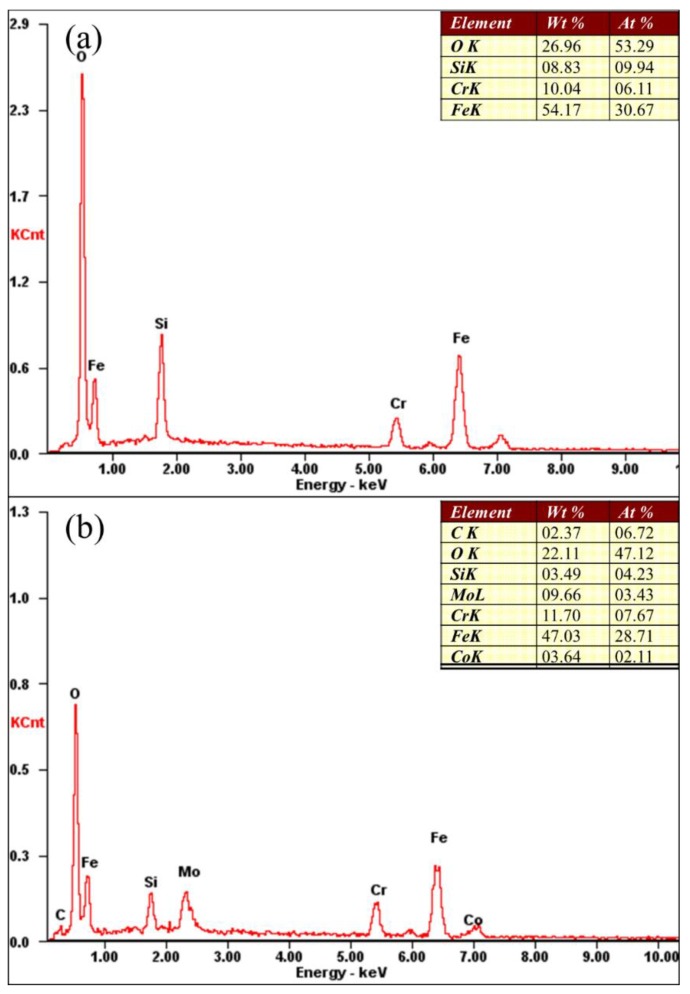
EDS results of different zones in Figure 10: (**a**) Zone 1; (**b**) Zone 2.

Figure 10 shows the morphologies of the worn surfaces of the substrate and the coatings obtained at different scanning speeds. Deep furrows with a peeling of large oxide patches could be observed in the worn surface of the substrate. The worn substrate surface (given in Figure 11a) had a composition of mainly Fe, O, Cr, Si elements using the EDS spectrum results in Figure 11. Significantly, a higher silicon content indicates that silicon was transferred from the upper sample to the substrate, indicating that adhesive wear had occurred. It can be inferred that the main wear mechanism of the substrate was adhesive wear and oxidative wear. At the scanning speed of 6 mm/s, there was only a small number of light grooves and a small amount of oxide particles on the surface of the cladding layer (Figure 10b). Its main wear mechanism was abrasive wear. The formation of the high hardness amorphous phase gave it the highest wear resistance. At the scanning speed of 8 mm/s, the surface of the cladding layer was uniform, and deeper grooves than those at 6 mm/s could be observed (Figure 10c). The wear resistant was high because of the presence of the high hardness ${\mathrm{Mo}}_{2}C,\;M_{7}C_{3}$ carbides in the cladding layer. This is mainly due to abrasive wear, and the carbide particles falling off from the cladding layer may be the source of the abrasive. When the scanning speed increased to 10 mm/s, the wear resistance of the cladding layer was notably reduced due to its lower hardness when compared to those obtained at scanning speeds of 6 mm/s and 8 mm/s (Figure 10d). Large oxides (of which the EDS results are given in Figure 11b) and furrows appeared on the worn surface, which indicated that adhesion and oxidative wear occurred on the cladding layer surface.

## 4. Conclusions

An Fe-based amorphous coating was successfully prepared by laser cladding of powder with a chemical composition of ${\mathrm{Fe}}_{46.8}{\mathrm{Mo}}_{22.7}{\mathrm{Cr}}_{13.6}{\mathrm{Co}}_{7.6}C_{4.8}B_{2.3}Y_{1.2}{\mathrm{Si}}_{1.0}$ (wt.%) on a 3Cr13 stainless steel substrate. The influences of laser scanning speed on the microstructure and properties of the coatings were investigated. The main results can be summarized as follows:

(1) The laser cladding layers exhibited three distinct microstructures at various scanning speeds. At the scanning speed of 6 mm/s, the cladding layer was a mixture of amorphous and crystalline regions. For a scanning speed of 8 mm/s, the cladding layer was mainly composed of a block grain structure. For a scanning speed of 10 mm/s, the cladding layer was composed entirely of dendrites. The dilution ratios were more dominant than the scanning speed in determining the resultant microstructures.
(2) Compared with the hardness of the substrate of 200 ${\mathrm{HV}}_{0.2}$, the cladding layers had an extremely high hardness of about 1300 ${\mathrm{HV}}_{0.2}$ at scanning speeds of 6 mm/s and 8 mm/s, and a lower high hardness of 700 ${\mathrm{HV}}_{0.2}$ at the scanning speed of 10mm/s.
(3) The wear resistance of the cladding layers was much higher than that of the substrate, and the wear resistance of the cladding layers could be improved by using a lower scanning speed due to the formation of high hardness wear resistant phases.

## Figures and Tables

**Table 1:** Chemical composition of the 3Cr13 stainless steel (wt.%).

Element	C	Mn	Ni	Si	P	S	Cr	Fe
wt.%	0.26–0.35	≤1.00	≤0.60	≤1.00	≤0.035	≤0.03	12.00–14.00	Balance

**Table 2:** Laser processing parameters.

Laser Power (W)	Scanning Speed (mm/s)	Powder Feeding Rate (g/min)	Heat Input (J/mm)	Overlapping Rate (%)
1000	6	20	166.7	30
1000	8	20	125.0	30
1000	10	20	100.0	30

**Table 3:** Average geometric parameters and dilution rates for the different scanning speeds.

Scanning Speed (mm/s)	Width of the Cladding Layer (μm)	Height of the Cladding Layer (μm)	Depth of the Molten Substrate (μm)	Dilution Rate
6	2139.2	906.9	68.3	7.0%
8	2070.7	749.6	120.6	13.9%
10	1965.8	409.2	230.6	36.0%
